# Clinical evaluation of multiplex PCR for the differential diagnosis of major pathogenic mycobacteria in East China

**DOI:** 10.1097/MD.0000000000045906

**Published:** 2025-11-14

**Authors:** Tingting Fang, Lijun Peng, Zhaodong Li, Huanyu Li, Hao Li, Long Cai

**Affiliations:** a Clinical Laboratory Center, Hangzhou Red Cross Hospital, Hangzhou, Zhejiang, China.

**Keywords:** multiplex PCR, *Mycobacterium tuberculosis*, nontuberculous mycobacteria, pathogenic mycobacteria, typing

## Abstract

Distinguishing between *Mycobacterium tuberculosis* (MTB) and nontuberculous mycobacteria infections remains clinically challenging due to their drastically different treatment regimens, underscoring the critical need for rapid and accurate mycobacterial identification to guide effective patient management. This study aimed to develop a multiplex polymerase chain reaction (PCR) assay for the detection and typing of major pathogenic mycobacteria – including MTB, *Mycobacterium avium complex* (MAC), *Mycobacteroides chelonae* – *Mycobacteroides abscessus* group (MCAG), and *Mycobacterium kansasii* – and to evaluate its diagnostic performance. The assay was designed with species-specific primers targeting 5 key genes: 16S rRNA, RD9, ITS (internal transcribed spacer) region-MAC, ITS region-MCAG, and *mkan-rs12360*. Its feasibility and lower limit of detection (LOD) were validated using 6 standard mycobacterial strains; diagnostic accuracy was further assessed using 68 clinical isolates and 130 clinical sputum samples, with whole-genome sequencing and targeted next-generation sequencing serving as the respective gold standards for isolates and sputum specimens. The multiplex PCR assay exhibited high specificity for the target genes and primers: for standard strains, the LOD ranged from 0.7 to 5.0 pg/μL; for artificially simulated sputum samples, the LOD was 5.6 to 26.7 pg/μL for single infections and 7.1 to 43.9 pg/μL for mixed infections. Against whole-genome sequencing, the assay achieved 100% detection rate and accuracy for clinical isolates. When compared with targeted next-generation sequencing for clinical sputum samples, the detection rates for single infections of MTB, MAC, MCAG, and *M kansasii* were 26%, 75%, 56.5%, and 50%, respectively, with an overall detection rate of 51.2% and 100% diagnostic accuracy. This multiplex PCR assay enables rapid and accurate identification of major pathogenic mycobacteria in clinical isolates; however, its sensitivity limitations – evidenced by relatively low detection rates in clinical sputum samples, especially mixed infections – highlight the need for further optimization with more sensitive probe-based technologies to enhance clinical applicability.

## 1. Introduction

The genus *Mycobacterium* encompasses various species, including the *Mycobacterium tuberculosis* complex (MTBC), *Mycobacterium leprae*, and nontuberculous mycobacteria (NTM). Tuberculosis (TB), caused by *Mycobacterium tuberculosis* (MTB), continues to be a major global health concern, remaining one of the top 10 leading causes of death worldwide. In 2021, an estimated 10.6 million people were afflicted with TB, resulting in 1.6 million deaths, and the TB incidence rate increased by 3.6% compared to 2020.^[1]^ The incidence and prevalence of NTM infections are also on the rise worldwide.^[2]^ In developed countries, the infection rate of NTM even surpasses that of TB, making it a “neglected global threat.”^[3]^ So far, there are over 200 confirmed NTM species, most of which are conditionally pathogenic or nonpathogenic bacteria, nevertheless, some NTMs exhibit strict or potential pathogenicity.^[3,4]^ Diseases caused by NTM can present with clinical symptoms and imaging changes like TB. According to statistics, without proper pathogen identification, up to 10% of cases may be misdiagnosed as TB and receive TB treatment.^[5,6]^ However, most NTMs display inherent resistance to commonly used anti-TB drugs, necessitating different treatment regimens than those for TB,^[7]^ and even different NTM species require distinct treatment approaches.^[8–10]^ Therefore, accurate and rapid identification and diagnosis of *Mycobacterium* species is crucial for precise treatment.

The distribution of pathogenic NTM strains has regional differences, but the most common strains are mainly *Mycobacterium avium* complex (MAC, including *Mycobacterium intracellulare*, *Mycobacterium chimera*, and *M avium*, etc), *Mycobacteroides chelonae*-*Mycobacteroides abscessus* group (MCAG, including *M abscessus*, *Mycobacterium massiliense*, and *M chelonae*, etc), and *M kansasii.*^[11–14]^ In the Zhejiang region of eastern China, these predominant pathogenic NTM collectively account for 93% of all NTM clinical isolations.^[15]^

The diagnostic process for mycobacterial infections encompasses smear microscopy, culture, and molecular detection. Smear microscopy is a simple and cost-effective technique, but it exhibits a low positive rate and cannot differentiate between MTB and NTM. Culture, while valuable, also has a low positive rate and requires a longer testing cycle, often exceeding 4 weeks.^[16,17]^ With the rapid advancements in molecular biology technology, an increasing number of molecular detection methods are being employed for *Mycobacterium* strain identification. The WHO recommended Xpert MTB/RIF (Cepheid, Sunnyvale) is widely used in China and can provide resistance results for MTBC and rifampicin within 2 hours, but cannot detect NTM.^[18]^ The CapitalBio Mycobacterium RT-polymerase chain reaction (PCR) Detection Kit (CapitalBio RT-PCR, CapitalBio Technology, China) developed in China can distinguish MTBC and NTM infections using dual PCR technology, but cannot distinguish NTM species.^[19]^ Other methods include PCR restriction fragment length polymorphism analysis,^[20–22]^ gene sequencing,^[23,24]^ and microarray technology.^[25]^ However, these approaches demand expensive equipment and highly skilled technicians, making them primarily available in large hospitals or specialized TB centers.^[2]^ For routine laboratories in grassroots hospitals, there is an imminent need for the development of a strain classification and identification method that is more accessible in terms of equipment requirements and ease of operation.

In this study, multiple PCR and agarose gel electrophoresis were used to identify and diagnose the main pathogenic mycobacteria prevalent in the Zhejiang region of eastern China, including MTB, MAC, MCAG, and *M kansasii* were evaluated for their diagnostic efficacy in clinical isolates and sputum samples.

## 2. Materials and methods

### 2.1. Collection of strains and clinical sample

Strain collection: 64 clinical *Mycobacterium* strains (13 species) and 4 non-mycobacterial clinical pathogenic strains were collected from Hangzhou Red Cross Hospital between June and December 2022. All these *Mycobacterium* were validated through whole-genome sequencing, 4 types of non-mycobacteria validated by mass spectrometry. Additionally, our laboratory maintained 6 standard strains (Table S1, Supplemental Digital Content, https://links.lww.com/MD/Q621).

Clinical sample collection: based on clinical diagnosis of mycobacterial infection as the inclusion criterion, the DNA of sputum samples confirmed by targeted next-generation sequencing (tNGS) as positive for target mycobacteria from March 2021 to March 2023 was retrospectively collected from the TB laboratory of Hangzhou Red Cross Hospital. The diagnosis of all TB cases followed the “Chinese Clinical Guidelines for Tuberculosis Treatment” and TB treatment guidelines,^[26]^ the diagnosis of all NTM cases adhered to the “Diagnosis and Treatment Guidelines for Nontuberculous Mycobacterial Disease in China.”^[27]^

### 2.2. Development of multiplex PCR

#### 2.2.1. Main mycobacterium target gene selection

Main *Mycobacterium* target gene selection based on the bioinformatics analysis results, we have chosen 5 target genes (16S rRNA, RD9, internal transcribed spacer [ITS] region-MAC, ITS region-MCAG, and *mkan-rs12360*) for the detection of 4 clinically significant *Mycobacterium* species, 5 sets of primers were designed for these target genes.

#### 2.2.2. DNA extraction

Nucleic acid extraction of standard and clinical bacterial strains: initially, colonies in the logarithmic growth phase were selected from the Lowenstein–Jensen medium and transferred to a 1.5 mL EP tube containing glass beads. Subsequently, 50 μL of normal saline was added and the mixture was agitated for 10 minutes. Then, the mixture was incubated in a water bath at 95°C for 15 minutes. Ultimately, the extracted nucleic acids were stored at −80°C.

Clinical sample nucleic acid extraction: nucleic acids were extracted from clinical sputum samples according to the instructions of the CapitalBio Mycobacterium RT-PCR Detection Kit and stored at −80°C.

#### 2.2.3. Multiplex PCR amplification and result interpretation

The total reaction volume for multiplex PCR is 10 μL, consisting of 1.8 μL of sample DNA, 5 μL of SYBR Green I (2×), and 3.2 μL of mixed primers. The positive control contains 1000 pg/μL of DNA from the standard bacterial strain *H37Rv*, while the negative control consists of RNase-free water. DNA amplification can be performed using a standard PCR machine, the following PCR program is employed: initial denaturation at 95°C for 10 minutes; followed by 30 cycles of denaturation at 96°C for 45 seconds, annealing at 61.5°C for 45 seconds, and extension at 72°C for 40 seconds; and a final extension step at 72°C for 10 minutes. The PCR products are then identified through 4% agarose gel electrophoresis, and the DNA bands are visualized using ultraviolet light from a gel imager. Interpretation rules for the results are presented in Figure S1, Supplemental Digital Content, https://links.lww.com/MD/Q621.

#### 2.2.4. Specific validation of target genes and primers

Six standard strains of *Mycobacterium* were utilized for the initial verification of the species specificity of target genes. To confirm primer specificity and assess potential mutual interference, the DNA from each of the 6 standard strains, each with a nucleic acid concentration of 1200 pg/μL, was combined in equal proportions for multiple PCR amplifications and subsequent result analysis.

#### 2.2.5. Verification of the limit of detection (LOD)

DNA from 6 standard bacterial strains was serially diluted in RNase-free water to form gradients ranging from 0.1 pg/μL to 500 pg/μL. To assess the effects of sputum inhibitors on multiplex PCR, the diluted strain DNA was mixed at a 1:4 ratio with negative sputum extract (from non-mycobacterial infection patients), the final concentration gradients of each strain were formed to range from 5 pg/μL to 200 pg/μL. For evaluating primer interference in mixed infections, DNA from all 6 strains was combined, diluted, and mixed at a 1:4 ratio with negative sputum extract, The final concentration gradients of each species, including the standard strain *H37Rv*, MAC, MCAG, and *M kansasii*, were set to range from 5 pg/μL to 100 pg/μL. All concentration gradients and negative controls (RNase-free water and negative sputum extract) were tested 20 times. The limit of detection (LOD) was defined as the lowest concentration detectable with 95% probability.

### 2.3. Clinical evaluation of multiplex PCR

#### 2.3.1. Verification of clinical isolates

To determine whether multiple PCR can accurately differentiate the target strain from other species, we conducted additional strain specificity validation on 68 clinical isolates, including 20 MTB strains, 44 NTM strains, and 4 non-mycobacteria strains. All clinical isolates were subjected to multiple PCR amplification and result analysis at a concentration of 1000 pg/μL.

#### 2.3.2. Evaluation of accuracy of clinical sputum samples

Evaluate the accuracy of 130 clinical samples using tNGS as a reference standard. tNGS procedure was developed as follows: multiplex PCR amplicons were prepared using a Ligation Sequencing Kit (SQK-LSK109; ONT, Oxford, UK) and a Native Barcoding Kit (EXP-NBD196; ONT). End-prep and native barcode ligation to amplicons were performed according to the Native Barcoding Kit protocol using a 300-ng sample diluted in 70 μL of nuclease-free water. Subsequently, adapter ligation and cleaning steps were carried out using a NEB ligation kit and AMPure XP magnetic beads (Beckman Coulter, Brea), respectively, resulting in a final adapter-ligated DNA library containing 100 ng of DNA. The pooled library was loaded into a flow cell (FLO-MIN106 R9.4, ONT), and DNA sequencing was performed using a MinION Mk1C sequencing device (ONT). Real time sequencing data were collected using the MinKnow v3.6.5 software (ONT). After completing the sequencing run, the Nanopore raw data (fast5) were base called using Guppy v4.5.2 software (ONT). Trimmed amplicon sequences were compared with mycobacterial reference sequences to obtain results for the MTB and NTM gene analysis. This analysis was conducted using an nBLAST-based bioinformatics pipeline created by Dian Diagnostics Co., Ltd. (Hangzhou, China).

### 2.4. Reagents and instruments

We employed 2× Cham Q Universal SYBR qPCR Master Mix (Nanjing VAzyme Biotech Co., Ltd, China) and primers (Hangzhou You Kang Biotechnology Co., Ltd, China). The instrumentation included a nucleic acid concentration tester, Nano Drop 2000 (Thermo Scientific, Waltham), a PCR machine, CFX96 Deep Well TM Real Time System (Bio-Rad Laboratories, Hercules), and a gel imager (TANON 1600, Shanghai, China). The gel image processing software is Photoshop.

## 3. Result

### 3.1. Selection and specificity verification of multiple PCR target genes

This study selected specific targets for Pan *Mycobacterium*, MTB, MAC, MCAG, and *M kansasii* (Table 1). The 16S rRNA gene was widely used for the species identification of mycobacteria,^[28]^ in this study, the 16S rRNA primers referred to the previously published sequences to distinguish Pan *Mycobacterium* from non-mycobacteria.^[29]^ The RD9 gene has been confirmed to specifically target MTB.^[30]^ The ITS region of the 16S-23S rRNA gene has been identified as a potentially suitable target for differentiating closely related *Mycobacterium* species,^[31]^ MAC and MCAG were targeted using specific sequences within the intergenic spacer region ITS region in this study. The target for *M kansasii* was a highly conserved sequence in the *mkan-rs12360* region.^[29,32]^ Preliminary validation was conducted on standard strains of 6 main pathogenic mycobacteria, and the experimental results showed that specific target genes can correctly identify all target strains. All strains produced 16S rRNA and corresponding target gene amplifiers, while mixed strain samples produced 16S rRNA and all target gene amplifiers (Fig. S2, Supplemental Digital Content, https://links.lww.com/MD/Q621).

**Table 1:** Target strain and amplification fragment size corresponding to target genes.

Set	Genetic target	Target organism(s)	Primer sequences (5′–3′)	Size (bp)
1	16S rRNA	Pan mycobacterial species	F:GAGATACTCGAGTGGCGAAC	506
			R:CAACGCGACAAACCACCTAC	
2	RD9	*M tuberculosis*	F:GTGTAGGTCAGCCCCATCC	358
			R:GTGTGGATTCCGTGGGCG	
3	ITS region-MAC	*M avium* complex	F:ATGATTGCCAGACACACTATTG	164
			R:ATTACACATTTCGATGAACGC	
4	ITS region-MCAG	*M chelonae-M abscessus* group	F:TAAGGAGCACCATTTCCCAG	128
			R:CGACGTTTTGCCGACTACC	
5	*mkan-rs12360*	*M kansasii*	F:ACAACGCATCTCATGACCACTC	208
			R:AACAGCACCTCGGCGAACT	

ITS region-MAC, the specific targets of *M avium* complex in ITS genes; ITS region-MCAG, the specific target of *M chelonae-M abscessus* group in ITS gene.

ITS = internal transcribed spacer, MAC = *Mycobacterium avium* complex, MCAG = *Mycobacteroides chelonae-Mycobacteroides abscessus* group.

### 3.2. Multiple PCR sensitivity verification

**Figure 1.**
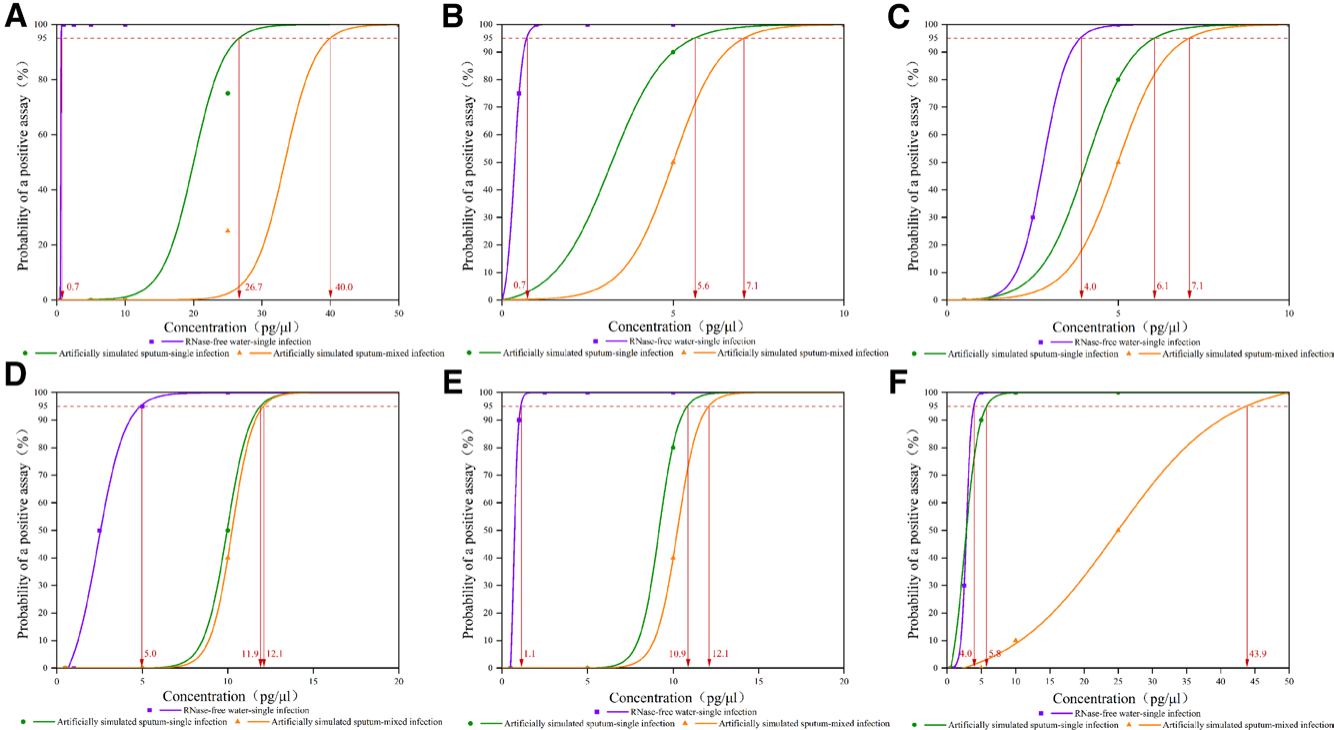
Multiplex PCR sensitivity analysis. (A) LOD of *H37Rv*; (B) LOD of *M avium*; (C) LOD of *M intracellulare*; (D) LOD of *M chelonae*; (E) LOD of *M abscessus*; (F) LOD of *M kansasii*. LOD = limit of detection, PCR = polymerase chain reaction.

LOD values of the multiplex PCR were determined using gradient-diluted DNA from 6 standard strains of *Mycobacterium*, the lowest concentration with a detection rate of 95% is the final LOD. The sensitivity range of PCR analysis for all detected *Mycobacterium* strains is as follows: 0.7 pg/μL *H37Rv* DNA, 0.7 pg/μL *M avium* DNA, 4.0 pg/μL *M intracellulare* DNA, 5.0 pg/μL *M chelonae* DNA, 1.1 pg/μL *M abscessus* DNA, and 4.0 pg/μL *M kansasii* DNA. The sensitivity range of single infectious in artificially simulated sputum samples is: 26.7 pg/μL *H37Rv* DNA, 5.6 pg/μL *M avium* DNA, 6.1 pg/μL *M intracellulare* DNA, 11.9 pg/μL *M chelonae* DNA, 10.9 pg/μL *M abscessus* DNA, and 5.8 pg/μL *M kansasii* DNA. The sensitivity range of mixed infection in artificially simulated sputum samples is: 40.0 pg/μL *H37Rv* DNA, 7.1 pg/μL MAC DNA, 12.1 pg/μL MCAG DNA, and 43.9 pg/μL *M kansasii* DNA (Fig. 1).

### 3.3. Verification of clinical isolates by multiple PCR

Further validation of bacterial species specificity was conducted on 17 types of clinical isolates, totaling 68 strains. The experimental results showed that all PCR products of *Mycobacterium* only had specific bands and no nonspecific bands. Non-mycobacteria showed no bands in the multiplex PCR. A total of 60 strains of 6 target mycobacteria (MTB, *M avium*, *M intracellulare*, *M chelonae*, *M abscessus*, and *M kansasii*,) and 3 subspecies (*M paraintracellulare, M chimera*, and *M massiliense*) were detected with a detection rate of 100% and an accuracy rate of 100%. The other 4 nontarget NTM only detected 16S rRNA of the Pan *Mycobacterium* genus gene, which was consistent with the expected results, with 100% accuracy. The 4 non-mycobacteria strains did not amplify any target genes, which was consistent with the expected results (Table 2).

**Table 2:** Multiple PCR specificity validation for different species.

Species	No	Target gene					Results interpretation	Accuracy(%)
		16S rRNA	RD9	*mkan-rs12360*	ITS-MAC	ITS-MCAG		
MTB								
* M tuberculosis*	20	+	+	–	–	–	MTB	100
Target NTM								
* M avium*	5	+	–	–	+	–	MAC	100
* M intracellulare*	5	+	–	–	+	–	MAC	100
* M paraintracellulare*	5	+	–	–	+	–	MAC	100
* M chimera*	5	+	–	–	+	–	MAC	100
* M chelonae*	5	+	–	–	–	+	MCAG	100
* M abscessus*	5	+	–	–	–	+	MCAG	100
* M massiliense*	5	+	–	–	–	+	MCAG	100
* M kansasii*	5	+	–	+	–	–	*M kansasii*	100
Other NTM								
* M simiae*	1	+	–	–	–	–	Other NTM	100
* M parascrofulaceum*	1	+	–	–	–	–	Other NTM	100
* M triplex*	1	+	–	–	–	–	Other NTM	100
* M lentiflavum*	1	+	–	–	–	–	Other NTM	100
Other species								
* Cryptococcus-neoformans*	1	–	–	–	–	–	–	100
* Aspergillus fumigatus*	1	–	–	–	–	–	–	100
* Staphylococcus aureus*	1	–	–	–	–	–	–	100
* Klebsiella pneumoniae*	1	–	–	–	–	–	–	100

ITS-MAC, ITS region-MAC; ITS-MCAG, ITS region-MCAG; +, generate corresponding amplification products; –, no corresponding amplification products; other NTM = NTM other than the target strain; –, not detected.

ITS = internal transcribed spacer, MAC = *Mycobacterium avium* complex, MCAG = *Mycobacteroides chelonae-Mycobacteroides abscessus* group, MTB = *Mycobacterium tuberculosis*, NTM = nontuberculous mycobacteria.

### 3.4. Evaluation of clinical applicability of multiple PCR

**Figure 2.**
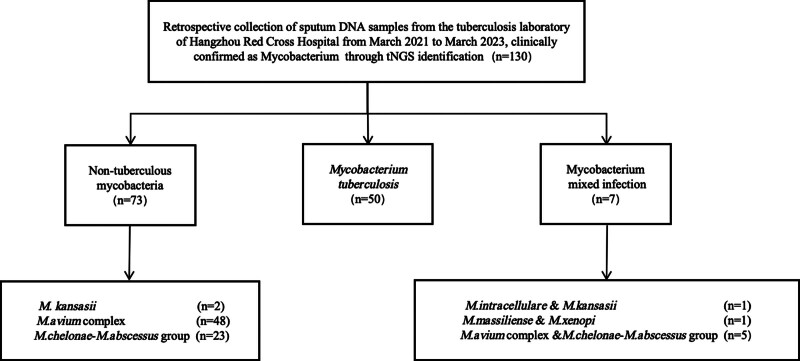
Sample inclusion flowchart. tNGS = targeted next-generation sequencing.

A total of 130 clinical sputum samples were included in this study (Fig. 2). The tNGS results showed 50 cases (38.5%) of MTB, 48 cases (36.9%) of MAC, 23 cases (17.7%) of MCAG, 2 cases (1.5%) of *M kansasii*, and 7 cases of NTM mixed infection samples. Using tNGS results as a reference standard, the detection rates of MTB, MAC, MCAG, and *M kansasii*, by multiplex PCR were 26%, 75%, 56.5%, and 50%, respectively. The total detection rate was 51.2%, with an accuracy of 100% (Table 3). Among the 7 mixed infection samples, there was 1 case that was consistent with the expected results (*M massiliense* and *Mycobacterium xenopi*), 1 case that was partially consistent (*M intracellulare* and *M kansasii*), and 5 samples that were not detected (MAC and MCAG; Table 4). The quantitative PCR data detected by tNGS simultaneously showed that among 50 clinical sputum samples with MTB, 13 MTB samples detected by multiplex PCR had CT values of 17.84 ± 3.7, 37 TB samples not detected by multiplex PCR had CT values of 25.03 ± 3.4, and 5 mixed infection samples not detected had CT values of 27.78 ± 1.1 (data not shown).

**Table 3:** Comparison of multiple PCR and tNGS results in single infection.

Species	tNGS	Multiplex PCR		
	No. identified	No. correct detection	Detection (%)	Accuracy (%)
*M tuberculosis*	50	13	26	100
*M avium* complex	48	36	75	100
*M chelonae*-*M abscessus* group	23	13	56.5	100
*M kansasii*	2	1	50	100
Total	123	63	51.2	100

PCR = polymerase chain reaction, tNGS = targeted next-generation sequencing.

**Table 4:** Detection and analysis of multiple PCR in multiple infections.

	tNGS	Multiplex PCR						Detection status
S/N	Multiple infections	16S rRNA	RD9	*mkan-rs12360*	ITS-MAC	ITS-MACG	Results interpretation
4	*M intracellulare* & *M abscess*	–	–	–	–	–	–	Not detected
13	*M intracellulare* & *M abscess*	+	–	–	–	–	–	Not detected
30	*M intracellulare* & *M kansasii*	+	–	+	–	–	*M kansasii*	Partially detected
46	*M intracellulareand* & *M abscess*	+	–	–	–	–	–	Not detected
61	*M intracellulare* & *M abscess*	–	–	–	–	–	–	Not detected
65	*M massiliense* & *M xenopi*	+	–	–	–	+	MCAG	Accurately detected
129	*M intracellulare* & *M avium* & *M abscess*	–	–	–	–	–	–	Not detected

&, indicating multiple infections; ITS-MAC, ITS region-MAC; ITS-MCAG, ITS region-MCAG; +, generate corresponding amplification products; –, no corresponding amplification products; –, undetected or invalid results.

ITS = internal transcribed spacer, MAC = *Mycobacterium avium* complex, MCAG *= Mycobacteroides chelonae-Mycobacteroides abscessus* group, PCR = polymerase chain reaction, S/N = sample sequencing number, tNGS = targeted next-generation sequencing.

## 4. Discussion

In this study, we developed a multiplex PCR technique combined with agarose gel electrophoresis detection method. This method targets the main pathogenic mycobacteria in eastern China, including MTB and 3 types of NTM (MAC, MCAG, and *M kansasii*). According to the research and statistics of the Zhejiang Provincial Center for Disease Control and Prevention in eastern China, the prevalence distribution of pulmonary NTM in Zhejiang Province is as follows: MAC (67.97%); MCAG (16.52%); *M kansasii* (8.17%). These 3 mains pathogenic NTM account for approximately 93% of all clinically isolated NTM strains.^[15]^ According to each target gene, a total of 5 pairs of primers were designed. Except for 16S rRNA, all primers were newly designed and optimized for the development of multiplex PCR detection in this study.

The validation results of 6 standard strains of *Mycobacterium* and 68 clinical isolates showed that the detection results of all strains were consistent with the expected results, with a detection rate and accuracy of 100%. This indicates that the 5 target sequences have good species specificity and can correctly distinguish the 4 main pathogenic mycobacteria. Although only 3 mains pathogenic NTM populations can be detected, and no species or subspecies can be distinguished, this can cover over 93% of NTM infection cases in the Zhejiang region, meeting the primary needs. Due to the inclusion of targets for Pan *Mycobacterium* in the system, for Pan *Mycobacterium* infection samples other than the 4 target strains, the target 16S rRNA (506 bp) of Pan *Mycobacterium* will display as positive, while the 4 specific targets will display as negative. In such cases, we can use methods such as gene chips and tNGS for further identification.

In recent years, numerous studies and consensuses have shown that tNGS, which combines ultra-multiplex PCR amplification with high-throughput sequencing, exhibits good consistency with the culture method in identifying mycobacteria, and even demonstrates a higher detection rate. It can directly detect dozens to hundreds of known pathogenic microorganisms and their virulence and/or drug resistance genes in clinical samples and has significant advantages in detecting mixed infections. Although tNGS is not the gold standard, it has shown the potential to replace the culture method in mycobacterial identification, particularly in terms of timeliness, sensitivity, and specificity. Therefore, we used the tNGS test results as the reference standard to evaluate the accuracy of the multiplex PCR test results.^[33–39]^ Among the 130 clinically diagnosed sputum samples confirmed by tNGS, 123 cases had a single infection, 63 cases detected by multiplex PCR. Although the detection rate for single infections was not high, the accuracy of the multiplex PCR results compared to tNGS was 100%. Additionally, tNGS results showed that there were 7 cases of mixed infections in the clinical samples, mainly involving different species of NTM (such as MAC and MCAG). Unfortunately, within these samples presenting mixed infections, merely 1 case was precisely detected, and another one was partially detected.

Multiplex PCR has shown good results in clinical isolates, but the detection rate in clinical samples is relatively low, especially for MTB samples and mixed infections. We believe the reasons are as follows: Firstly, agarose gel has lower resolution and sensitivity, and the nucleic acid needs to be amplified to a certain amount before bands can be observed visually. The LOD of this method for standard strains is relatively high, ranging from 0.7 to 5.0 pg/μL. Both standard strains and clinical strains have high concentrations of extracted nucleic acids, allowing for detection. Secondly, there are inhibitory substances present in clinical sputum samples. Literature reports indicate that unknown inhibitory substances in sputum samples can lead to false negative results in PCR detection, with a range from 10% to 26%.^[40]^ The higher LOD of artificially simulated sputum samples compared to the LOD of standard strains in our study also supports this point. Thirdly, some clinical sputum samples may have low target nucleic acid concentrations below the lower LOD of this method. We performed statistical analysis on quantitative PCR data obtained simultaneously with tNGS. Among the 50 clinical sputum samples tested for MTB, there is an average difference of 7 CT values between the detected and undetected samples in the multiplex PCR assay, indicating a relatively lower DNA concentration in the undetected samples (data not shown). Fourthly, tNGS’s high sensitivity may cause false positives from trace contaminated DNA, leading to result deviations.^[39]^ In conclusion, to improve the detection rate in clinical sputum samples, it is necessary to lower the lower LOD and improve the sensitivity of the method.

This study has certain limitations. First, the number of included strains and samples is relatively small, which fails to fully validate the detection ability of multiplex PCR in clinical strains and clinical samples (especially those with mixed infections). Second, the number of NTM species verified in clinical samples is relatively limited, particularly for *M kansasii*. Third, the clinical evaluation of multiplex PCR uses tNGS as the reference standard instead of the gold standard culture method. Due to its high sensitivity, tNGS may yield false positives, leading to deviations in the results. In subsequent research, we will include more clinical strains and samples for validation. In addition to using the culture method as a reference standard, we will also combine multiple methods as evaluation criteria for the new method.

Melting curve analysis is effective for distinguishing *Mycobacterium* species but can be affected by nonspecific amplification, potentially leading to inaccuracies.^[41]^ The CapitalBio RT-PCR, which uses TaqMan probe-based dual PCR technology, can differentiate between MTB and NTM infections but not between NTM species. In contrast, the multiplex PCR method employed in this study can identify 4 major pathogenic mycobacterial species, but it has lower sensitivity compared to the TaqMan probe technology. TaqMan probes can detect DNA as low as 10 fg.^[42]^ In another study of ours that employed TaqMan probe technology, a LOD of 11.7 to 360.0 CFU/mL was achieved.^[43]^ While this technology significantly enhances sensitivity, it requires expensive equipment. Our next step is to replace the agarose gel method with nucleic acid mass spectrometry and combine this method with multiplex PCR. Based on the results of this study, our goal is to develop a more sensitive and low-cost detection method, to improve the overall detection rate of clinical samples – especially those with mixed infections.

## 5. Conclusions

In summary, this method demonstrates high specificity in targeting genes and primers, allowing for the rapid and accurate identification of major pathogenic mycobacteria in clinically isolated strains, offering low cost, minimal equipment requirements, simple operation, and a short detection cycle. However, due to the limitations of methodological sensitivity, the detection rate in clinical sputum samples is inadequate, especially in samples with mixed infections. In the next step, a more sensitive detection method will be developed based on this study.

## Acknowledgments

We greatly appreciate the provision of strains and clinical samples by the Tuberculosis Laboratory at Hangzhou Red Cross Hospital.

## Author contributions

**Conceptualization:** Long Cai.

**Data curation:** Tingting Fang, Lijun Peng.

**Funding acquisition:** Zhaodong Li.

**Methodology:** Tingting Fang, Lijun Peng.

**Project administration:** Hao Li.

**Supervision:** Long Cai.

**Software:** Huanyu Li.

**Validation:** Tingting Fang.

**Visualization:** Tingting Fang, Lijun Peng.

**Writing** – **original draft:** Tingting Fang.

**Writing** – **review & editing:** Tingting Fang, Long Cai, Lijun Peng.

## Supplementary Material


