# Supplementary material for: Clinical evaluation of multiplex PCR for the differential diagnosis of major pathogenic mycobacteria in East China
Source: Medicine (Baltimore). 2025 Nov 14;104(46):e45906. doi: 10.1097/MD.0000000000045906 (PMC12622619; doi:10.1097/MD.0000000000045906)

| Supplementary Table 1 List of Bacteria and Standard strains used in this study | | |
| --- | --- | --- |
| Bacterial species | No | Strains |
| **Mycobacterial species** | | |
| *H37Rv* | 1 | Standard strain(ATCC27294) |
| *M.avium* | 1 | Standard strain(ATCC700898) |
| *M.intracellulare* | 1 | Standard strain(ATCC13950) |
| *M.chelonae* | 1 | Standard strain(ATCC35752) |
| *M.abscessus* | 1 | Standard strain(ATCC19977) |
| *M.kansasii* | 1 | Standard strain(ATCC12478) |
| *M. tuberculosis* | 20 | Clinical isolates (Confirmed by WGS) |
| *M.avium* | 5 | Clinical isolates (Confirmed by WGS) |
| *M.intracellulare* | 5 | Clinical isolates (Confirmed by WGS) |
| *M.paraintracellulare* | 5 | Clinical isolates (Confirmed by WGS) |
| *M.chimaera* | 5 | Clinical isolates (Confirmed by WGS) |
| *M. chelonae* | 5 | Clinical isolates (Confirmed by WGS) |
| *M. abscessus subsp. abscessus* | 5 | Clinical isolates (Confirmed by WGS) |
| *M. abscessus subsp. massiliense* | 5 | Clinical isolates (Confirmed by WGS) |
| *M.kansasii* | 5 | Clinical isolates (Confirmed by WGS) |
| *M. simiae* | 1 | Clinical isolates (Confirmed by WGS) |
| *M. parascrofulaceum* | 1 | Clinical isolates (Confirmed by WGS) |
| *M. triplex* | 1 | Clinical isolates (Confirmed by WGS) |
| *M. lentiflavum* | 1 | Clinical isolates (Confirmed by WGS) |
| **Non-mycobacterial species** | | |
| *Cryptococcus -neoformans* | 1 | Clinical isolates (Confirmed by Mass Spectrometry) |
| *Aspergillus fumigatus* | 1 | Clinical isolates (Confirmed by Mass Spectrometry) |
| *Staphylococcus aureus* | 1 | Clinical isolates (Confirmed by Mass Spectrometry) |
| *Klebsiella pneumoniae* | 1 | Clinical isolates (Confirmed by Mass Spectrometry) |

WGS, Whole Genome Sequencing.

**Supplementary Figure 1** Result interpretation flowchart.


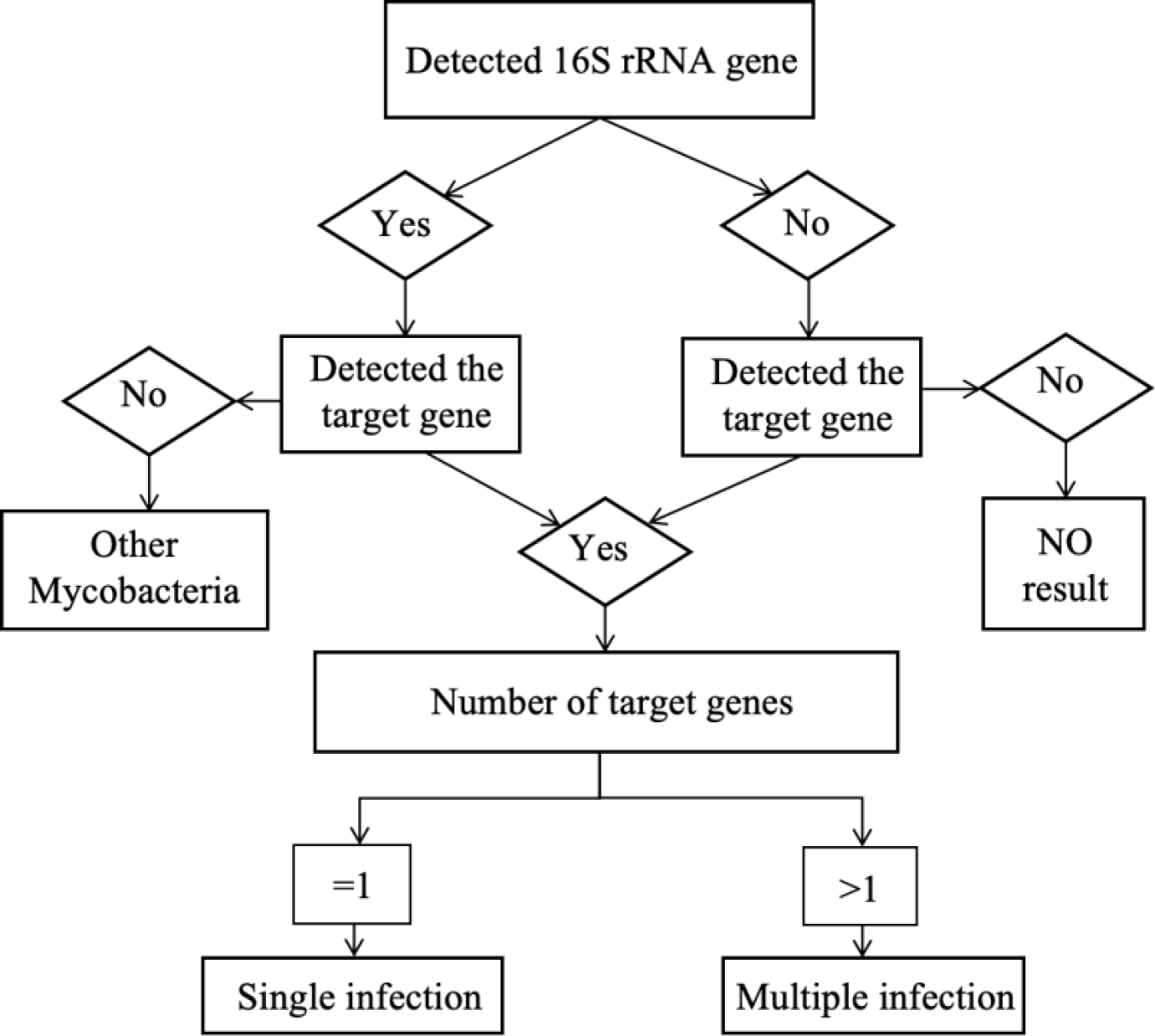


**Supplementary Figure 2** Multiple PCR species specificity validation results for six standard strains of Mycobacterium. M: Marker; 1: *H37Rv*; 2: *M. kansasii*; 3: *M. avium*; 4: *M. intracellulare*; 5: *M. chelonae*; 6: *M. abscessus*; 7-8: Results of a mixture of six standard bacterial strains; 9: NTC, No Template Control.


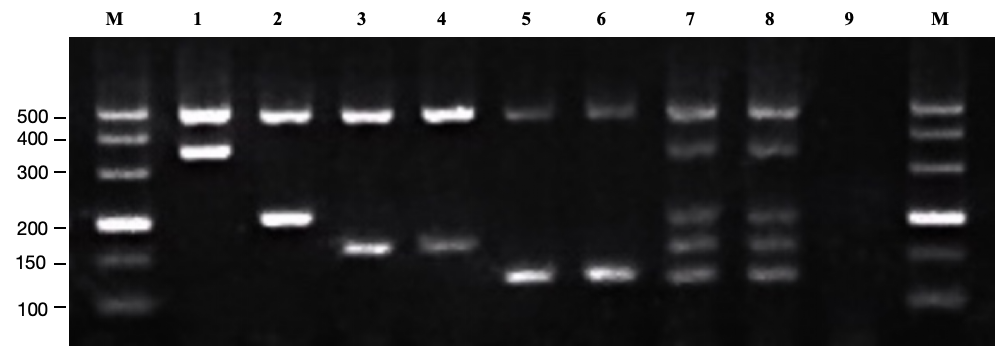

Supplement: Supplementary file 1 [file medi-104-e45906-s001.docx]
